# Biological networks in Parkinson’s disease: an insight into the epigenetic mechanisms associated with this disease

**DOI:** 10.1186/s12864-017-4098-3

**Published:** 2017-09-12

**Authors:** Paulami Chatterjee, Debjani Roy, Malay Bhattacharyya, Sanghamitra Bandyopadhyay

**Affiliations:** 1 Department of Biophysics, Bose Institute, Acharya J.C. Bose Centenary Building, P-1/12 C.I.T. Scheme VII M, Kolkata, 700054 India; 2 Department of Information Technology, Indian Institute of Engineering Science and Technology, Shibpur, Botanic Garden, Howrah, PO 711103 India; 3 Machine Intelligence Unit, Indian Statistical Institute, 203 B.T. Road, Kolkata, 700018 India

**Keywords:** Parkinson’s Disease, Gene co-expression network, Gene regulatory network, Feed forward loop, Long non-coding RNA, microRNA, SNPs, Epigenetics

## Abstract

**Background:**

Parkinson’s disease (PD) is the second most prevalent neurodegenerative disorders in the world. Studying PD from systems biology perspective involving genes and their regulators might provide deeper insights into the complex molecular interactions associated with this disease.

**Result:**

We have studied gene co-expression network obtained from a PD-specific microarray data. The co-expression network identified 11 hub genes, of which eight genes are not previously known to be associated with PD. Further study on the functionality of these eight novel hub genes revealed that these genes play important roles in several neurodegenerative diseases. Furthermore, we have studied the tissue-specific expression and histone modification patterns of the novel hub genes. Most of these genes possess several histone modification sites those are already known to be associated with neurodegenerative diseases. Regulatory network namely mTF-miRNA-gene-gTF involves microRNA Transcription Factor (mTF), microRNA (miRNA), gene and gene Transcription Factor (gTF). Whereas long noncoding RNA (lncRNA) mediated regulatory network involves miRNA, gene, mTF and lncRNA. mTF-miRNA-gene-gTF regulatory network identified a novel feed-forward loop. lncRNA-mediated regulatory network identified novel lncRNAs of PD and revealed the two-way regulatory pattern of PD-specific miRNAs where miRNAs can be regulated by both the TFs and lncRNAs. SNP analysis of the most significant genes of the co-expression network identified 20 SNPs. These SNPs are present in the 3′ UTR of known PD genes and are controlled by those miRNAs which are also involved in PD.

**Conclusion:**

Our study identified eight novel hub genes which can be considered as possible candidates for future biomarker identification studies for PD. The two regulatory networks studied in our work provide a detailed overview of the cellular regulatory mechanisms where the non-coding RNAs namely miRNA and lncRNA, can act as epigenetic regulators of PD. SNPs identified in our study can be helpful for identifying PD at an earlier stage. Overall, this study may impart a better comprehension of the complex molecular interactions associated with PD from systems biology perspective.

**Electronic supplementary material:**

The online version of this article (10.1186/s12864-017-4098-3) contains supplementary material, which is available to authorized users.

## Background

Parkinson’s disease (PD) is one of the well-reported neurodegenerative disorders, only second to the Alzheimer’s disease (AD), throughout the world [1]. The primary pathology of PD is the loss of dopaminergic neurons in the substantia nigra with Lewy bodies (intracytoplasmic inclusion deposits of aggregated alpha-synuclein and ubiquitin protein, and damaged nerve cells) [2, 3].

A good number of studies have been performed to identify the causative factors and molecular markers of PD. Several previous studies have pointed out the role of different genes in this disease [4]. Gene expression profiling analysis has identified differentially expressed genes in PD [5]. Besides, differential expression of several microRNAs (miRNAs) has also been associated with the pathophysiology of several neurodegenerative diseases [6, 7] including PD [8]. Study of gene regulatory networks has emerged as an important approach for computational analyses of diseases [9]. However, limited previous studies have tried to understand the association of both of these (miRNAs and mRNAs) PD markers in the context of biological networks. In order to gain a proper understanding of this disease, one needs to study the detailed regulatory network involving genes, miRNAs and transcription factors (TFs). A thorough examination of regulatory networks can help us to identify the key genes or miRNAs as well as different network motifs associated with a disease. These network motifs, in turn, provide us several important aspects of a disease progression.

Previous studies have indicated the role of epigenetic modifications in the development of neurodegenerative diseases including Parkinson’s Disease and Alzheimer’s Disease (AD) [10, 11]. Epigenetics refers to the meiotically and mitotically heritable changes in gene expression that does not involve changes to the DNA sequence [12]. Interpretation of epigenetic profiling leads to the identification of changes in gene expression responsible for the disease progression. There are three distinct yet highly interrelated mechanisms of epigenetic regulation - DNA methylation, Histone modifications and non-coding RNA-based mechanisms [13]. Epigenetic changes can be influenced by several factors including age, environment, lifestyle and disease state [13]. Recent systematic review on neurodegenerative disease, investigated epigenetic marks in PD and identified the most consistently reported methylation genes and histone modifications associated with PD [14].

Studies have revealed that non-coding RNAs such as miRNAs (~22 nt long) and long non-coding RNAs (lncRNAs) (>200 nt long), play crucial roles in epigenetic pathways and gene silencing. The function of miRNAs involves binding to a specific sequence in the 3′ UTR of a gene and inhibiting the expression of that gene. Thus, miRNAs act as cellular post-transcriptional regulators. The miRNA profiling of PD samples offers insight into the molecular mechanism of PD progression and several miRNAs have been implicated in PD pathogenesis [8, 15, 16].

The function of lncRNAs involves diverse cellular processes, such as chromatin remodeling, cell cycle regulation and several developmental processes [17]. It can influence the post-transcriptional regulation by interfering with the miRNA pathways, by acting as competing endogenous RNAs (ceRNAs) [18]. lncRNAs possess miRNA response elements (MRE) or miRNA binding sites in them. This enables lncRNAs to act as miRNA sponges to control the availability of endogenous miRNA for binding to their target mRNAs and subsequently reducing the repression of these target mRNAs [18]. lncRNAs are implicated in neurodegenerative processes, including AD and Huntington’s disease (HD) [19, 20]. However, very little is known about the association of lncRNAs in PD [21].

Single base alteration in the gene sequence or single nucleotide polymorphism (SNP) can affect the phenotypes either by altering the amount of protein produced or by changing the type of protein produced [22]. SNPs are believed to cause differences between individuals, such as susceptibility to diseases [23]. There are numerous SNPs present in the human genome [24]. These are considered as invaluable markers and potentially powerful tools for both genetic research and applications in practice [25]. Several studies have identified SNPs associated with complex diseases, which in turn serve as a potential marker for diagnosis [26]. A recent miRNA-related SNP analysis study identified SNPs as independent prognostic markers for the survival in small cell lung cancer patients [27]. However, very few such studies have been performed for PD. A recent genome-wide association study identified significant association between bone marrow stromal cell antigen 1 SNP and increased risk of PD which is enhanced by environmental factors [28]. SNP analysis or genotyping of PD patients can be helpful to identify this disease at an earlier state. Besides single base alteration in the miRNA binding sites can give us important information about the mode of regulation of regulatory factors in this disease. It is believed that more and more genetic studies combined with machine learning and statistical methods will be required in near future to investigate the underlying molecular signature of a disease [29].

With the increase of transcriptomic data, novel system biological approaches are required that can explore the complex molecular interactions associated with a disease. In this study, we have analyzed gene co-expression network based on a PD microarray dataset. Two regulatory networks were built from the highly co-expressed genes. The mTF-miRNA-gene-gTF regulatory network involves microRNA Transcription Factor (mTF), microRNA (miRNA), gene and gene Transcription Factor (gTF) whereas, long noncoding RNA (lncRNA) mediated regulatory network involves miRNA, gene, mTF and lncRNA. mTF-miRNA-gene-gTF regulatory network identified a novel feed-forward loop. lncRNA-mediated regulatory network identified novel lncRNAs of PD and revealed the two-way regulatory pattern of PD-specific miRNAs where miRNAs can be regulated by both the TFs and lncRNAs. SNP analysis of the most significant genes of the co-expression network identified 20 SNPs. Thus, our study provides important insight into the epigenetic mechanism (lncRNA, miRNA, histone modification) associated with PD. Moreover, SNPs identified in our study can be helpful for identifying PD at an earlier stage.

## Results

**Fig. 1:**
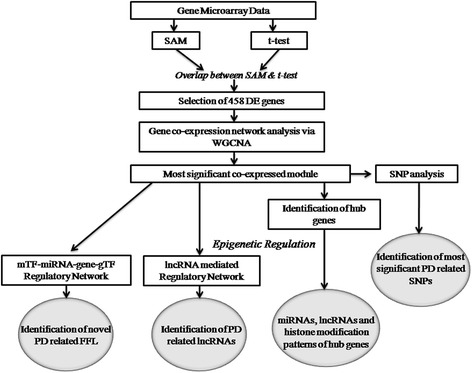
Workflow of the methodology used in our study

Figure 1 depicts the workflow of our analysis.

### Differentially expressed gene selection

#### SAM

We identified the differentially expressed (DE) genes between PD and control patients by applying the Significance Analysis of Microarray (SAM) [30]. In chip A, SAM identified 1518 DE genes at FDR value 0.19% and tail strength 44.1%. Among the 1518 DE genes 293 genes were positive (upregulated) and 1225 were negative (downregulated). In chip B, SAM identified 673 DE genes at FDR value 0.11% and tail strength 37.6%. Among the 673 differentially expressed genes, 91 genes were positive (upregulated) and 582 were negative (downregulated).

#### t-test

With the t-test analysis, a much higher number of DE genes were found than with SAM. Results identified 4797 and 3120 DE genes in chip A and chip B respectively at *p*-value 0.05 or 95% confidence level.

The common DE genes found by both SAM and t-test were considered as the most significant DE gene sets and these were used for further study. In chip A, 521 genes were found to be commonly DE in both SAM and t-test whereas, in chip B, 130 genes were found to be commonly DE in both SAM and t-test (Table 1). Out of the 521 and 130 genes from chip A and chip B only 458 genes in chip A and 105 genes in chip B were annotated.

**Table 1:** DE genes separately identified by SAM and t-test and DE genes commonly identified by both

	DE genes (SAM)	Annotated DE genes (SAM)	DE genes (t-test)	Annotated DE genes (t-test)	DE genes (common to SAM & t-test)	Annotated DE genes (common to SAM & t-test)
chip A	1518	1417	4797	4436	521	458
chip B	673	372	3120	1606	130	105

### Enrichment analysis of the DE genes

The DE genes found from SAM and t-test were both annotated via EASE (Expression Analysis Systematic Explorer) [31]. The shared 458 DE genes of chip A obtained from SAM and t-test were then subjected to enrichment analysis in FatiGO (Table 2) [32]. Results of the enrichment analysis of identified several neurodegenerative disease pathways as the most significant over representative KEGG pathways such as Parkinson’s disease pathway (hsa05012), Huntington disease (hsa05016) and Alzheimer’s disease (hsa05010) (Table 3). This also signifies the importance of this gene set in the context of the PD-specific study. The 105 DE genes in chip B were not associated with any significant terms in FatiGo. Therefore DE genes of chip B were not considered for further analysis. The 458 DE genes of chip A were considered as the significant gene set for further study and were termed as common DE gene set of chip A.

**Table 2:** FatiGO analysis results of the common DE genes of chip A and chip B obtained from SAM and t-test

	No of significant term associated with the 458 DE genes of chip A	No of significant term associated with the 105 DE genes of chip B
GO Biological Process	85	0
GO Cellular Component	30	0
GO Molecular function	18	0
KEGG Pathway	7	0

**Table 3:** Highly significant KEGG pathways associated with the common 458 genes of chip A identified in FatiGO analysis

Term	Name	*P* value	Genes
hsa05012	Parkinson’s Disease Pathway	3.82E-09	SNCA,UBE2J1,NR4A2,NDUFA9,ATP5A1,UCHL1,VDAC3,NDUFA5,ATP5B,ATP5D,CYCS,ATP5O,CYC1,PINK1,NDUFAB1,ATP5G3
hsa00190	Oxidative phosphorylation	3.50E-07	NR4A2,NDUFA9,ATP5A1,ATP6V0D1,NDUFA5,ATP6V1B2,ATP5B,ATP6V0C,ATP6V1C1,ATP5D,ATP5O,CYC1,NDUFAB1,ATP5G3
hsa05016	Huntington disease	3.64E-05	NDUFA9,ATP5A1,VDAC3,NDUFA5,AP2M1,ATP5B,DCTN2,ATP5D,CYCS,ATP5O,CYC1,NDUFAB1,ATP5G3
hsa05010	Alzheimer’s disease	9.90E-05	SNCA,NDUFA9,ATP5A1,CALM3,NDUFA5,ATP5B,ATP5D,CYCS,ATP5O,CYC1,NDUFAB1,ATP5G3
hsa04142	Lysosomes	1.63E-04	SORT1,LAMP2,NPC1,IDS,AP3M2,NR4A2,AP3B2,ATP6V0D1,ATP6V0C,LAPTM4B
hsa03050	Proteasome	6.61E-04	PSME3,PSMD12,PSMA1,PSMD8,PSMD1,PSMB2
hsa04722	Neurotrophin signaling pathway	7.01E-04	NFKBIA,SORT1,MAGED1,CALM3,NGFRAP1,YWHAZ,YWHAB,HRAS,ARHGDIA

### Co-expression network construction and analysis

On the basis of the co-expression pattern, WGCNA (please refer to the methods section) divided the 458 common DE genes into six modules (turquoise, blue, brown, yellow, green and red containing 266, 56, 43, 42, 25 and 25 mRNAs). FatiGO analysis revealed that out of the six WGCNA modules Turquoise module was the most significant co-expressed module (Additional file 1: Table S1A and B).

#### Topological analysis of the WGCNA module and identification of hub genes

**Fig. 2:**
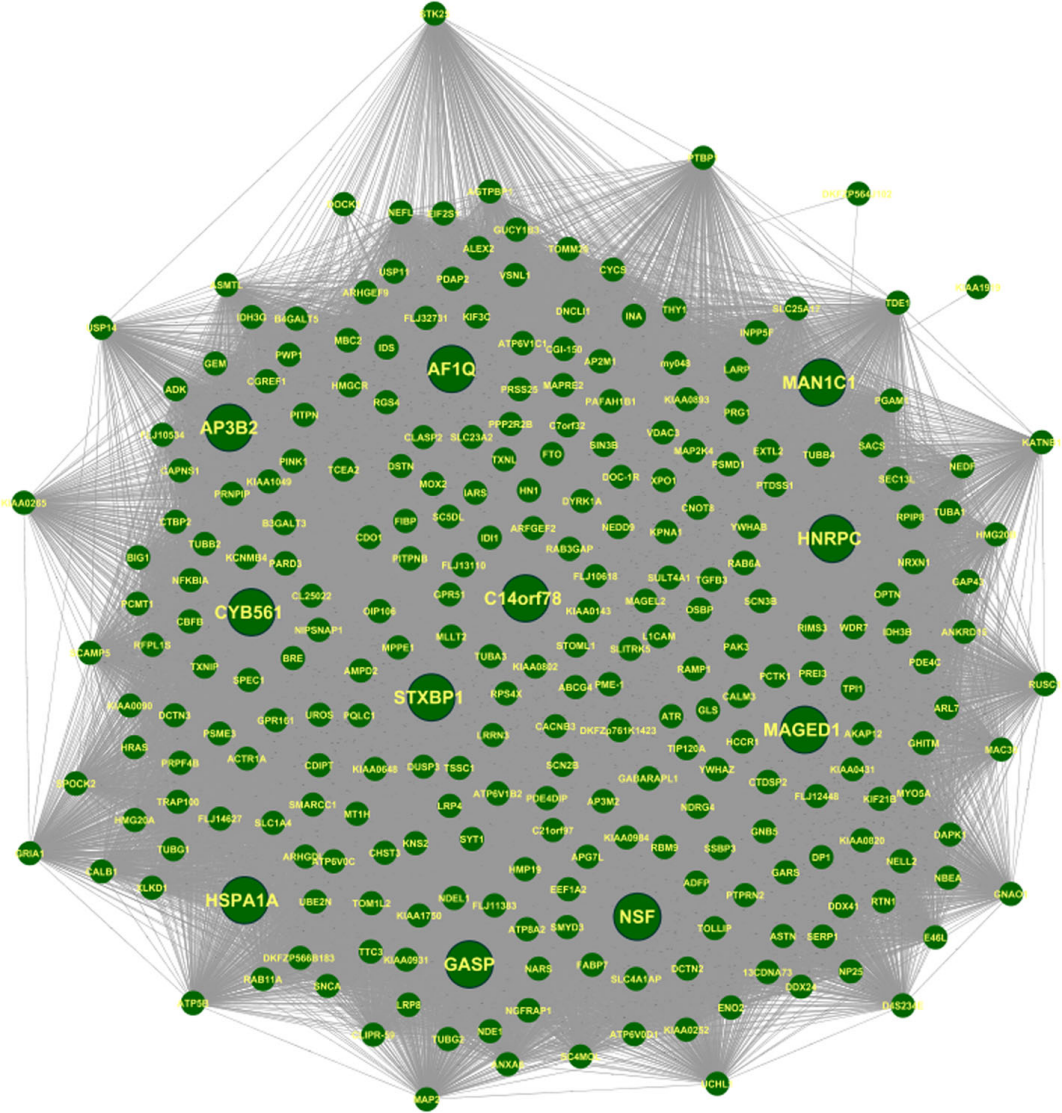
Gene co-expression Network of the most significant co-expressed module (Turquoise module) obtained from WGCNA. Green nodes represent genes and edges represent co-expression relationship. 11 Hub genes are represented by larger node size

We analyzed two centrality measures - degree and betweenness centrality (BC) in tYNA [33]. We sorted the 266 genes according to their degree or connectivity. The degree represents the number of connections or edges of a particular node [34], whereas BC quantifies the flow of information through a node in the network. It specifies how a node influences the communication among other nodes [35]. In our study, the 266 genes exhibited a varied degree distribution with the highest degree of 262 and lowest degree of 1. The average degree value was found to be 217.63 with standard deviation 52.74. We found that highest BC value was 391.50 and the lowest was 0 with an average of 24.95 and standard deviation 33.71. We chose the top 8 nodes (i.e. top 3% of the total nodes) with highest degree value as High Connectivity (HC) hub nodes. AP3B2, MAGED1, NSF, STXBP1, CYB561, AF1Q, C14ORF78 and GASP were identified as HC hub genes (Additional file 2: Table S2). Interestingly these 8 HC nodes were assigned with low BC values. Surprisingly we found three nodes with high BC values but low degree value. Although these nodes have low connectivity, they might be important regarding information flow. Therefore, we identified these as High BC low connectivity (HBLC) hub nodes. HNRPC, MAN1C1 and HSPA1A were identified as HBLC hub genes (Additional file 2: Table S2). Figure 2 shows the gene co-expression network of the turquoise module with the 11 hub genes. Out of the 11 hubs, three hubs (NSF, HSPA1A and CYB561) were already found to be associated with PD. The remaining eight novel hub genes (MAGED1, AP3B2, STXBP1, AF1Q, GASP, C14ORF78, MAN1C1, HNRPC) were further studied for their association in PD.

### Epigenetic regulation of the hub genes

In order to identify the probable epigenetic regulation of the hub genes, histone modification data for eight hub genes (Table 4) were retrieved from HHMD [36]. Table 4 shows that all the eight hub genes were associated with several histone modification sites. Further study identified the experimentally validated non-coding RNA mediated regulation of hub genes (Table 5) [37–40]. It was found that four out of the eight hub genes were associated with miRNAs already known in PD. Moreover, most of these hub gene associated miRs were in turn regulated by lncRNAs.

**Table 4:** Histone modification patterns (obtained from HHMD) of novel hub genes with respect to the already known histone modification sites in neurodegenerative diseases

Novel hub genes	Official symbol	RefSeq ID	Histone modification sites already known in neurodegenerative diseases				
			H3K27	H3K4	H3K9	H3K9/H4K20	H4R3
MAGED1	MAGED1	NM_006986	√	√	√	√	√
AP3B2	AP3B2	NM_004644	√	√	√	√	√
STXBP1	STXBP1	NM_001032221	√	√	√	√	√
AF1Q	MLLT11	NM_006818	√	√	√	√	√
GASP	GPRASP1	NM_014710	√	√	√	√	√
C14ORF78	AHNAK2	NM_138420	√	√	√	√	√
MAN1C1	MAN1C1	NM_020379	√	√	√	√	√
HNRPC	HNRNPC	NM_004500	√	√	√	√	√

**Table 5:** Regulatory non-coding RNAs associated with the novel hub genes identified in our study

Novel hub genes	miRNAs associated with novel hub genes	lncRNAs associated with the miRNAs
MAGED1	hsa-miR-3942–5p	
	hsa-miR-4703-5p	
	hsa-miR-3157-5p	
	hsa-miR-3188	
	hsa-miR-4649-3p	
	hsa-miR-3200-5p	
	hsa-miR-1252	
	hsa-miR-4777-5p	
	hsa-miR-760	
	hsa-miR-4474-3p	
	hsa-miR-4709-5p	
	hsa-miR-421	n339122
	hsa-miR-505	
	hsa-miR-4704-3p	
	hsa-miR-4252	
	hsa-miR-3120-3p	
	hsa-miR-3148	
	hsa-miR-4457	
	hsa-miR-4801	
	hsa-miR-4731-3p	
	hsa-miR-548o	
	hsa-miR-4762-3p	
	hsa-miR-450b-5p	
	hsa-miR-1323	
AP3B2	hsa-miR-221	n339827
	hsa-miR-222	
STXBP1	**hsa-miR-9**	
AF1Q	**hsa-let-7b**	n339682, XIST, n410735, n410470, n408209, n410533, n410111, n411752, n381104, n333512, n345604, RP11–139H15.1, n407908
	hsa-miR-1-2	
	hsa-miR-1-1	
	hsa-miR-155	
	**hsa-miR-16-1**	
	**hsa-miR-16-2**	
	**hsa-miR-30a**	n336002, n409199, RP11-46 M12.1
	**hsa-miR-30b**	
	**hsa-miR-30c-1**	
	**hsa-miR-30c-2**	
	hsa-miR-30d	
	hsa-miR-30e	n340869, n409200
	**hsa-miR-29a**	
	**hsa-miR-29c**	
	**hsa-miR-29b**	n410507, n341043
GASP	hsa-miR-873	
	hsa-miR-4711-5p	
	hsa-miR-4642	
	hsa-miR-3065-5p	
	hsa-miR-3671	
	hsa-miR-4277	
	hsa-miR-888	
	hsa-miR-4727-5p	
C14ORF78	hsa-miR-195	
	**hsa-miR-16**	n340911, n409656, n409286, n324249, n409199, n410507, n409266, n410476, n407230, n340530, n407036
	hsa-miR-424	RP11-690G19.3, n410128, **XIST**, n342875, n381422, **n406658**, n340847, **n338391**, n407096, n342697, n382508, n407461, n339766
	**hsa-miR-15a**	n342249, n409656, n407055, n410632, n408096, n381271, n410476, n342731, n410126, n406625, n335593, n341454, n409264, n409159, n408379, n337715, n338629, n409761
	hsa-miR-497	
	hsa-miR-15b	n410890, n410438, n338345
MAN1C1	hsa-miR-93-5p	n341008, **n410211**, n408146
	hsa-miR-130b-3p	n406921, n337752, n382094, n342786, n337985, n333016, n410686, n410036, n340556, n406580, n385717, n324749, n333275, n340852
	hsa-miR-206	
	**hsa-miR-1**	
	hsa-miR-613	
HNRPC	hsa-miR-455-5p	

Already known PD-specific miRNAs are shown in bold

Four lncRNAs regulating both PD-specific miRNAs and miRNAs not previously known in PD are shown in bold (please refer Table 6 for more details)

### Regulatory network construction and analysis

#### mTF-miRNA-gene-gTF regulatory network

**Fig. 3:**
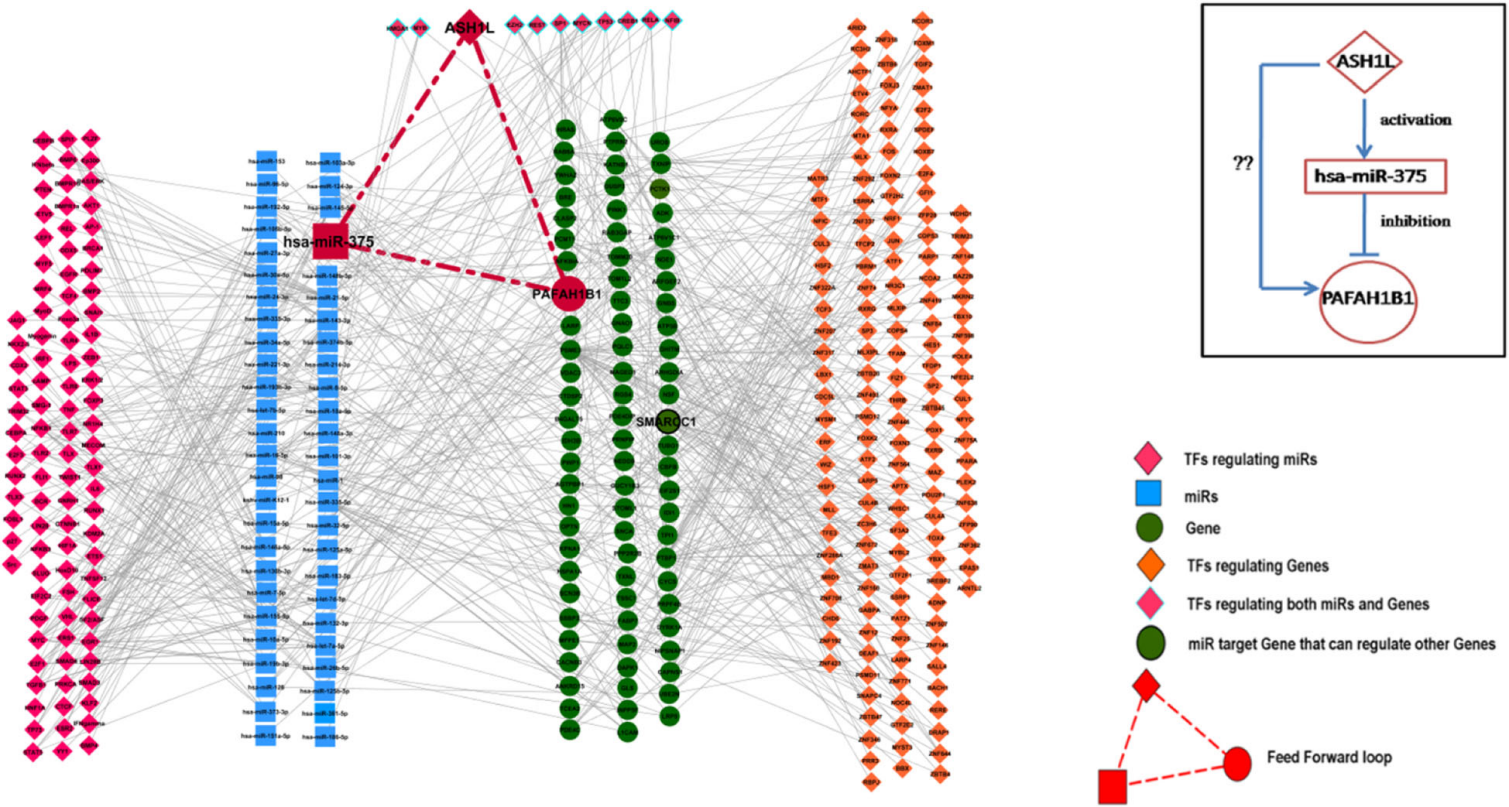
The four layered mTF-miRNA-gene-gTF Regulatory Network of the turquoise module. In this network, blue rectangular nodes represent miRNAs, green circular nodes represent genes, green circular node with black border represents gene that can regulate other genes as TF, diamond shaped magenta nodes represent mTFs, diamond shaped orange nodes represent gTFs, diamond shaped pink nodes with cyan borders represent the common TFs regulating both miRNAs and genes. The Feed-Forward Loop involving hsa-miRNA-375, gene PAFAH1B1 and TF ASH1L is also shown in the network

In order to get a view of the regulatory pattern of the turquoise module, we built a regulatory network comprising genes of the turquoise model and the TFs associated with these genes (gTFs) [41]. 160 gTFs were found to be associated with 81 genes of the turquoise module. It was found that PSME3 and PTBP1 are the genes, which are regulated by maximum gTFs, 31 and 25 gTFs respectively. Both of these genes were found to be involved in several cancers [42, 43]. PSME3 was found to be involved in Huntington’s Disease [44]. Besides, we found gTFs for hub gene, HSPA1A, MAGED1 and NSF.

two hundred twenty-six genes of the turquoise module were found to be associated with 51 experimentally validated miRNAs. ATP6V1C1, CBFB and PSME3 are the genes, which are regulated by a maximum number of miRNAs i.e. 7, 6 and 6 miRNAs, respectively. These 51 experimentally validated miRNAs were associated with 117 mTFs as obtained from TransmiR database [45]. By combing all these regulatory information, we constructed a mTF-miRNA-gene-gTF regulatory network (Fig. 3) which represents the four layers of complex regulatory interactions taking place within the most significant WGCNA module.

#### lncRNA-mediated gene regulatory network

Fifty-one miRNAs that were previously found to be associated with the genes of the turquoise module were further searched in the lncbase module of DIANA tools [40] to acquire information on the miRNA-lncRNA pair. Thirteen out of the 51 miRNAs were found to be PD-specific which were associated with 57 lncRNAs. These 13 miRs control 29 genes of the turquoise module and the 13 miRNAs are in turn controlled by 44 mTF. By combining all these regulatory information, we constructed a regulatory network involving the mTFs, lncRNA and genes (Table 6, Figure not Shown).

**Table 6:** lncRNA-mediated PD-specific regulatory network

PD-specific miRNA	miRNA associated TFs	miRNA associated lncRNAs	miRNA associated mRNAs
hsa-let-7a-5p	EIF2C2 (A), FSH (R), MYC (R), TRIM32 (A), E2F1 (A), E2F3 (A), LIN28 (R), LIN28B (R), NFKB1 (A), FLI1 (A), CEBPA	n410470	HRAS
hsa-let-7b-5p	EIF2C2 (A), MYC (R), TRIM32 (A), LIN28 (R), LIN28B (R), NFKB1 (A)	n339682, XIST, n410735, n410470	CBFB, IDI1, PSME3, SMARCC1
hsa-miR-103a-3p		n406427, n410632, n407114, n342319, n410010, n409184, n344659, TTC28-AS1, n338391, n339003, n342913, n407911, n409072, n409093	KPNA1, NSF
hsa-miR-125a-5p	EGR1, TLR2	n406658	DUSP3
hsa-miR-128		n410586, n406663, n408020	ATP6V1C1, GNB5, TXNIP, UBE2N
hsa-miR-15a-5p	c-myb (A), MYC (R), PRKCA (R), E2F1 (A), E2F3 (A), STAT5 (R)	n342249, n409656, n407055, n410632, n408096, n381271, n410476	HSPA1A, TPI1
hsa-miR-16-5p	MYC (R), E2F1 (A), E2F3 (A), STAT5 (R), NFKB1	n340911, n409656, n409286, n324249, n409199, n410507, n409266	ARHGDIA, CYCS, HSPA1A, KPNA1, L1CAM, LARP, NSF, PCMT1, PSME3, TPI1
hsa-miR-19b-3p	E2F1 (A), MYC (A), MYCN (A), NKX2–5 (A), TLX1 (A), TLX3 (A), ERS1 (A), STAT5 (A), SPI1 (R)	n336002, n341479, n409083, n339644, n333000, n339682, n333732, n337982, n340675	ATP6V1C1, LRP8, TOM1L2
hsa-miR-24-3p	BMP2 (A), TGFB1 (R), PDGF, RUNX2 (R), EGR1, ESR2 (A)	n340600, n409298	CTDSP2, NFKBIA, PSME3
hsa-miR-30a-5p	EGR1, ESR2 (R)	n336002, n409199, RP11-46 M12.1	ATP6V1C1, CBFB, KPNA1, NDE1, PRPF4B, YWHAZ
hsa-miR-34a-5p	MYC (R), TP53 (A), NR1H4 (R), CEBPA, NFKB1 (A), TP73 (A), SNAI1 (R), ZEB1 (R), E2F3	n406642, n385777, n408346, n341213, n408077, n410211, n335724	HSPA1A, KPNA1, LARP, SMARCC1, TXNIP
hsa-miR-7-5p	HoxD10 (A), SF2/ASF (A), FOXP3 (A)	n342007, n407908, n410669, n410711	GLS, PSME3, SNCA
hsa-miR-9-5p	IL1B (A), LPS (A), NFKB1 (A)(R), TLR2 (A), TLR4 (A), TLR7 (A), TLR8 (A), TLX (R), TNF (A), MYC (A), CREB1, REST	YTHDF3	OPTN

The mode of regulation of TF-miRNA pair is denoted by (A) = Activation, (R) = Repression

### SNP analysis of the most significant co-expressed module

#### Selection of biologically significant SNPs

SNPs corresponding to the 266 genes of the turquoise module were obtained from the online database SCAN [46]. We studied for those SNPs that are present at the 3’UTR of these genes. Using MirSNP database [47] (miRNAs usually bind at the 3’UTR of the target gene and controls the expression of that gene). 1525 miRNAs were found to be associated with these SNPs. Of these 1525 miRNAs, 82 miRNAs were found to be PD related. It was found that 140 SNPs were associated with these 82 miRNAs (*p*-value ≤10^−05^) (Additional file 3: Table S3). These 140 SNPs were then searched in the dbSNP database (http://www.ncbi.nlm.nih.gov/snp/) [48] for SNP sequence, chromosome locus, and gene corresponding to each of the SNPs (data not shown). It was found that these 140 SNPs were associated with 157 genes (out of the 266) of the turquoise module.

## Discussion

In our study, Co-expression network analyses revealed that out of the six WGCNA modules Turquoise module was the most significant co-expressed module. Enrichment analysis revealed that Parkinson disease pathway (hsa05012) is one of the over-representative pathways associated with this module (Additional file 1: Table S1A). Moreover, Epithelial cell signaling in *Helicobacter pylori* infection (hsa05120) appeared as one of the most significant KEGG pathways (Additional file 1: Table S1A). Previous studies have already reported that *H. pylori* infection is associated with PD [49]. Additional file 1: Table S1B depicts the highly Significant GO Biological Processes such as microtubule-based process (GO:0007017), synaptic transmission (GO:0007268), intracellular transport (GO:0046907), etc. associated with the genes of turquoise module.

Co-expression network yielded 11 hub genes based on their topological significance. Out of the 11 hubs, three hubs (NSF, HSPA1A and CYB561) were already found to be associated with PD. The remaining eight novel hub genes were further studied for their association in PD.

### Differential expression pattern of the hub genes

Table 7 represents the differential expression pattern of the eight novel hub genes obtained from the co-expression network. Most of the novel hub genes (MAGED1, AP3B2, STXBP1, AF1Q, GASP, C14ORF78, and MAN1C1) were down regulated in PD with respect to control, whereas, one hub gene (HNRPC) was up-regulated in PD with respect to control.

**Table 7:** Differential expression pattern and fold change of the eight co-expressed hub genes

Novel hub genes	Differential expression pattern	Fold Change (Obtained from SAM)
MAGED1	Down regulated	0.49
AP3B2	Down regulated	0.36
STXBP1	Down regulated	0.44
AF1Q	Down regulated	0.42
GASP	Down regulated	0.43
C14ORF78	Down regulated	0.29
MAN1C1	Down regulated	0.50
HNRPC	Up regulated	1.34

### Biological significance of hub genes

We further studied the biological significance of these eight novel hub genes. Table 8 represents the GO biological processes associated with the eight novel hub genes which shows the involvement of these genes in several PD-related processes such as protein transport, neurotransmitter release, synaptic transmission, etc. We found that a recent study has pointed out the role of MAGED1 in the central nervous system in both developmental and adult stages [50]. Studies have found reporting on the vesicle coat protein complex AP3B2 to have some neuron-specific functions such as neurotransmitter release [51, 52]. STXBP1 was found to be listed as an AD-specific marker in Genotator [53], Polysearch [54] and Pescador [55]. AF1Q is a retinoic acid target gene and reported to have an association with ovarian cancer disease [56]. GASP was found as a potential tumor marker for several cancers [57]. C14ORF78 was reported to be associated with calcium channel proteins of cardiomyocytes [58]. MAN1C1 was identified as a differentially expressed gene in PD blood samples. Transcriptome changes related to epigenetic modifications including chromatin remodeling and methylation was also studied for this gene in PD [59]. Protein products of HNRPC gene are associated with pre-mRNA processing and other aspects of mRNA metabolism and transport [60]. All these information validate our finding regarding the association of these genes as hubs in PD.

**Table 8:** GO Biological processes associated with the novel hub genes

Novel hub genes	GO Biological Process	GO Terms
MAGED1	Regulation of transcription, DNA-templated	GO:0006355
	Regulation of transcription from RNA polymerase	GO:0006357
	II promoter	
	Circadian regulation of gene expression	GO:0032922
	Regulation of circadian rhythm	GO:0042752
	Regulation of apoptotic process	GO:0042981
	Positive regulation of apoptotic process	GO:0043065
	Positive Regulation of MAP kinase activity	GO:0043406
	Negative regulation of transcription, DNA-templated	GO:0045892 GO:0045893
	Positive regulation of transcription, DNA-templated Neurotrophin TRK receptor signaling pathway	
AP3B2	Intracellular protein transport	GO:0006886
	Post-Golgi vesicle-mediated transport	GO:0006892
	Anterograde axon cargo transport	GO:0008089
	Anterograde synaptic vesicle transport	GO:0048490
STXBP1	platelet degranulation	GO:0002576
	energy reserve metabolic process	GO:0006112
	vesicle docking involved in exocytosis	GO:0006904
	synaptic transmission	GO:0007268
	neurotransmitter secretion	GO:0007269
	neuromuscular synaptic transmission	GO:0007274
	axon target recognition	GO:0007412
	regulation of synaptic vesicle priming	GO:0010807
	glutamate secretion	GO:0014047
	protein transport	GO:0015031
AF1Q	Positive regulation of apoptotic process	GO:0043065
	Positive regulation of transcription, DNA-templated	GO:0045893
	Positive regulation of mitochondrial depolarization	GO:0051901
	Positive regulation of release of cytochrome c from mitochondria	GO:0090200
	Extrinsic apoptotic signaling pathway	GO:0097191
	Intrinsic apoptotic signaling pathway	GO:0097193
GASP	Endosome to lysosome transport	GO:0008333
	G-protein coupled receptor catabolic process	GO:1,990,172
	G-protein coupled receptor catabolic process	GO:1,990,172
C14ORF78	Plasma membrane repair	GO:0001778
MAN1C1	Protein N-linked glycosylation	GO:0006487
	Protein N-linked glycosylation via asparagine	GO:0018279
	Post-translational protein modification	GO:0043687
	Cellular protein metabolic process	GO:0044267
HNRPC	mRNA splicing, via spliceosome	GO:0000398
	Osteoblast differentiation	GO:0001649
	RNA splicing	GO:0008380
	Gene expression	GO:0010467
	ATP-dependent chromatin remodeling	GO:0043044
	3′-UTR-mediated mRNA stabilization	GO:0070935

### Epigenetic regulation of hub genes

The epigenetic regulations of hub genes are shown in Tables 4 and 5. We have studied the association of experimentally validated miRNAs and lncRNAs with eight hub genes. It was found that four (STXBP1, AF1Q, C14ORF78, MAN1C1) out of the eight hub genes were regulated by PD-specific miRNAs. Interestingly, AF1Q was identified to be regulated by a maximum number of PD-specific miRNAs (10). It is evident from Tables 5 and 6 that four lncRNAs namely (XIST, n406658, n338391, n410211) are regulating both PD-specific miRNAs and miRNAs not previously known in PD.

We have studied the histone modification patterns of hub genes. Histone modification refers to the posttranslational modifications of the amino-terminal tails of histone proteins which upon modification affect the downstream molecular interactions, hence regulates the gene expression. Interestingly, we found several histone modification sites those are already known to be associated with several neurodegenerative diseases [61] present within these eight hub genes (Table 4).

### Identification of feed forward loop from mTF-miRNA-gene-gTF regulatory network

Analysis of regulatory network revealed the presence of an interesting FFL, where a TF regulates a miRNA and they both regulate a target gene (Fig. 3). We found such a FFL between the gene PAFAH1B1, hsa-miR-375 and TF ASH1L. TransmiR data indicated that hsa-miR-375 is activated by TF ASH1L. By combing the TransmiR and TarBase data, we found that ASH1L and hsa-miR-375 both regulate the expression of its target gene PAFAH1B1. Studies have found that ASH1L activates hsa-miR-375 and hsa-miR-375 inhibits its target PAFAH1B1. Interestingly, however, ASH1L has been found to be over-expressed in neuroblastoma cell line transfected with normal or mutated alpha-synuclein [62]. This indicates a possibility of higher expression of this TF in brain tissues of PD patients. Besides, studies have identified the association of miR-375 in gastric cancer, breast cancer, cervical cancer [63–65]. A recent study with AD patients has identified higher expression of this miRNA (has-miR-375) in patients than controls [66]. This information provides a link to the finding of upregulation of hsa-miR-375 by the TF ASH1L. It is possible that up-regulation of this miRNA in PD patients is responsible for the aberrant production of downstream target genes involved in the pathogenesis. Moreover, the FFL gene PAFAH1B1 has been listed in Genotator database as a responsible candidate gene in AD. PAFAH1B1 was found to be associated with epilepsy, schizophrenia, neuronal migration disorders, cerebellar hypoplasia, etc. nerve related diseases in GeneCards database (http://www.genecards.org/). Therefore, this can be considered as a validation of our findings in PD. Further study on this novel FFL can help us to understand the molecular biology of PD progression.

### Significance of lncRNA-mediated gene regulatory network

This network depicts an interesting functional module where a PD-specific miRNA is being regulated by both mTF (either activation or repression) and lncRNA, and this regulatory information is then conveyed to the gene in terms of post-transcriptional repression. Modes of regulations of 44 mTFs associated with 13 PD-specific miRNAs (out of 51 miRNA of the turquoise module) indicated that most of these interactions were ‘activation’ (Table 6). The regulation of hsa-miR-103a-3p of this network is noteworthy. It is not associated with mTFs but has a maximum number of lncRNAs (14 lncRNAs) associated with it (Table 6). This miRNA represses two genes, namely, KPNA1 and NSF. NSF is known to be involved in PD [53] whereas KPNA1 is known to be involved in several neurological disorders including autism and schizophrenia [67]. In contrast to the above findings, hsa-let-7a-5p and hsa-miR-9-5p each has one identified lncRNA (Table 6). These two miRNAs are in turn repress one gene each namely HRAS and OPTN. These two genes are known to be involved in PD [68, 69]. However, both of the miRNAs are associated with 11 and 12 mTFs respectively (Table 6). Since all the 57 lncRNAs of this regulatory network are associated with known PD-specific miRNAs, they might be important epigenetic regulators in PD that were not identified by previous studies. Moreover, the conservation scores of 57 lncRNAs indicate high conservations that strengthen the association of these lncRNAs with PD (Additional file 4: Table S4).

### Final screening and selection of 20 most significant SNPs associated with PD

One hundred forty SNPs were identified from the 157 co-expressed genes of the turquoise module. Out of these 157 genes, 18 genes were already known in PD. 20 SNPs are identified to be associated with 18 genes which in turn controlled by PD-specific miRNAs. This strengthens the association of these 20 SNPs in PD (Table 9). In order to find out the functional role of these 20 SNPs, we further analyzed them in F-SNP database (http://compbio.Cs.Queensu.Ca/F-SNP/) [70]. Table 10 Describes the functional category, allele and region of each SNPs. Interestingly 3 SNPs namely, rs535860, rs3814309 and rs3766286 are found to be classified as the ‘conserved’ functional category (predicted by PhastCons_8way and PhastCons_17way within F-SNP database) signifying a conserved functional role of these variations throughout the evolution. Furthermore, our study identified several SNPs associated with hsa-miR-375 involved in FFL of the regulatory network. Among them, SNP rs193223230 is present in the locus of an already known PD-related gene (YWHAZ) (Table 11). Therefore, hsa-miR-375 can be an important PD epigenetic biomarker in our study.

**Table 9:** 20 most significant SNPs in PD with their associated PD-specific miRNAs and genes

microRNAs	SNPs	Population	*p-value*	Chromosome	Gene
hsa-miR-34a-5p	rs3750625	YRI	5.00E-05	10:112,839,601	ADRA2A
hsa-miR-34b-5p	rs3750625	YRI	5.00E-05	10:112,839,601	
hsa-miR-34c-5p	rs3750625	YRI	5.00E-05	10:112,839,601	
hsa-miR-29b-2-5p	rs1697406	YRI	9.00E-05	1:21,904,267	ALPL
hsa-miR-9-5p	rs1697406	YRI	9.00E-05	1:21,904,267	
hsa-miR-1225-5p	rs535860	YRI	1.00E-05	11:117,159,878	BACE1
hsa-miR-661	rs535860	YRI	1.00E-05	11:117,159,878	
hsa-miR-647	rs13198420	CEU	7.00E-05	6:38,139,482	BTBD9
hsa-miR-661	rs12206712	CEU	8.00E-05	6:38,139,748	
hsa-miR-455-3p	rs2762934	YRI	1.00E-05	20:52,771,261	CYP24A1
hsa-miR-632	rs3814309	CEU	4.00E-06	1:110,277,403	GSTM3
hsa-miR-199b-5p	rs16843618	YRI	1.00E-05	2:210,595,820	MAP2
hsa-miR-663b	rs3766286	YRI	3.00E-05	1:31,344,250	SDC3
hsa-let-7a-3p	rs1050955	YRI	1.00E-05	7:100,782,460	SERPINE1
hsa-let-7b-3p	rs1050955	YRI	1.00E-05	7:100,782,460	
hsa-let-7f-1-3p	rs1050955	YRI	1.00E-05	7:100,782,460	
hsa-miR-612	rs7242	YRI	4.00E-05	7:100,781,445	
hsa-miR-224-5p	rs12281100	YRI	7.00E-05	11:36,506,773	TRAF6
hsa-miR-1226-3p	rs2242437	YRI	6.00E-07	19:1,065,563	HMHA1
hsa-miR-130a-5p	rs1042364	CEU	4.00E-05	4:100,045,574	ADH4
hsa-miR-29a-3p	rs1051881	YRI	9.00E-05	4:122,737,965	CCNA2
hsa-miR-29b-3p	rs1051881	YRI	9.00E-05	4:122,737,965	
hsa-miR-29c-3p	rs1051881	YRI	9.00E-05	4:122,737,965	
hsa-miR-1253	rs17085675	YRI	4.00E-05	5:95,727,664	PCSK1
hsa-miR-30b-3p	rs1045968	CEU	4.00E-05	16:29,826,365	PRRT2
hsa-miR-612	rs281437	YRI	4.00E-05	19:10,397,238	ICAM1
hsa-miR-663b	rs3829972	YRI	2.00E-05	12:6,929,018	CD4
hsa-miR-374a-5p	rs8067	YRI	1.00E-05	9:95,218,829	ASPN

**Table 10:** Functional Categories of the 20 most significant PD-related SNPs

SNPs	Functional Category	Allele	Region
rs3750625	transcriptional_regulation	C/A	3 prime UTR
rs1697406	transcriptional_regulation	A/G	3 prime UTR
rs535860	conserved	A/T	3 prime UTR
rs13198420	none	T/C	3 prime UTR
rs12206712	none	T/C	3 prime UTR
rs2762934	transcriptional_regulation	A/G	3 prime UTR
rs3814309	transcriptional_regulation, conserved	T/C	3 prime UTR
rs16843618	transcriptional_regulation	G/C	3 prime UTR
rs3766286	transcriptional_regulation, conserved	C/T	3 prime near gene
rs1050955	transcriptional_regulation	G/A	downstream
rs7242	transcriptional_regulation	T/G	3 prime UTR
rs12281100	none	A/C	downstream
rs2242437	none	C/G	upstream
rs1042364	protein_coding	A/G	3 prime UTR
rs1051881	protein_coding, splicing_regulation, transcriptional_regulation, post_translation	G/C	nonsynonymous
rs17085675	transcriptional_regulation	A/T	3 prime UTR
rs1045968	transcriptional_regulation	G/T	intron, 3 prime UTR
rs281437	transcriptional_regulation	C/T	3 prime UTR
rs3829972	transcriptional_regulation	A/G	3 prime UTR
rs8067	transcriptional_regulation	C/A	Regulatory region, 3 prime UTR

**Table 11:** SNP associated with the FFL miRNA and PD-related gene

microRNA	SNPs	Gene Sequence	Chromosome	Gene
hsa-miR-375	rs193223230	CTTAACAATTATGCTTGGATTGTTC **[A/G]** TGAAAATTTCATAAGACATTAAACA	8:101932002	YWHAZ

Changes in the sequence are shown in bold

## Conclusion

In this study, we have analyzed gene co-expression network, gene regulatory network, and lncRNA-mediated regulatory network based on a PD microarray dataset. The co-expression network, generated through WGCNA, identified eight novel hub genes based on their topological significance in the network. The biological significance and epigenetic regulations of hub genes indicated their involvement in PD-related processes. Analysis of the gene regulatory network (mTF-miRNA-gene-gTF) resulted in the identification of a novel FFL, the regulators of which are unidentified in PD. The lncRNA-mediated regulatory network provided important insight into the lncRNA-mediated regulation of known PD miRNAs. These lncRNAs might be important epigenetic regulators in PD those were not identified by previous studies. Moreover, 57 lncRNAs obtained from lncRNA-mediated regulatory network indicate high conservations that strengthen the association of these lncRNAs with PD. Four lncRNAs (XIST, n406658, n338391, n410211) were identified to be regulating both PD-specific miRNAs and miRNAs not previously known in PD. Moreover, SNP analysis identified 20 significant SNPs along with their associated genes and regulatory miRNAs. These SNPs can be considered as potential risk factors upon further validation. Out of these 20 SNPs, 3 SNPs, namely rs535860, rs3814309 and rs3766286 have conserved functional role throughout the evolution. Thus, findings of our study will be helpful for further PD clinical research and diagnostic purposes.

## Methods

Figure 1 depicts the workflow of our analysis.

### Microarray data collection

Microarray data generated by Affymetrix HG_U133 array sets (A and B chips) was downloaded from GEO Dataset Browser for data set GDS3128 and series GSE 8397 (from the link http://www.ncbi.nlm.nih.gov/gds/?term=GDS3128) [71]. The microarray data contains 94 samples (47samples from chipA and 47 samples from ChipB) taken from three brain regions Frontal Cerebral Cortex (FCC), Lateral Substantia Nigra (LSN) and Medial Substantia Niagra (MSN). A total of 15 samples were taken from MSN, 9 from LSN, 5 samples from frontal cerebral cortex. 8 medial nigra control samples and 7 lateral nigra control samples and 3 frontal cerebral cortex control samples were considered. The whole dataset was normalized with GCRMA (Gene Chip Robust Multi-Array Averaging) which performs background correction, probe level intensity calculation and summarization [72].

### Analysis of differential gene expression

To identify the most significant DE gene set from the microarray data, we performed both the SAM and t-test analysis. When we performed t-test and SAM, we did not get any differentially expressed genes for frontal cerebral cortex.

#### SAM

Significance Analysis of Microarray (SAM) [30] was used to identify the differentially expressed (DE) genes that are positively and negatively regulated genes among the control and disease samples. The test statistic of SAM is given by:

$$ {d}_i=\frac{r_i}{s_i+{s}_o} $$

Where *d*_*i*_ is the relative difference in gene expression, *r* is the linear regression coefficient of gene *i*, *s*_*i*_ is the standard error of *r* and *s*_*o*_ is a constant chosen to minimize the coefficient of variation of *d*_*i*_. Thus, SAM assigns a score to each gene on the basis of change in gene expression relative to the standard deviation of repeated measurements. In chip A, SAM identified 1518 DE genes at FDR value 0.19%. In chip B, SAM identified 673 DE genes at FDR value 0.11%.

#### t-test

We further performed paired two sample t-test to identify differentially expressed genes in chip A and chip B. 2-tailed t-test is a measure of the statistical significance of the dataset, in terms of a test statistic *t*, which is given by:

$$ t=\frac{\overline{x}-\overline{y}}{\sqrt{\frac{{s_x}^2}{n}+\frac{{s_y}^2}{m}}} $$

Where $\overline{x}$ and $\overline{y}$ are the sample means, *s*_*x*_ and *s*_*y*_ are the sample standard deviations, *n* and *m* are the sample sizes for two samples, *x* and *y*. Under the null hypothesis, this test returns the probability (*p*-value) of observing a value as extreme or more extreme of the test statistic. Probes corresponding to a portion of the genes showed significant changes in signal intensities in disease sample groups, as compared to the control. These genes were selected as DE genes. t-test analysis identified 4797 and 3120 DE genes in chip A and chip B respectively at *p*-value 0.05 or 95% confidence level.

### Construction of the gene co-expression network

The 458 common DE genes from the chip A were subjected to Weighted Gene co-expression Network Analysis (WGCNA) [73]. This correlation networking method deals with genes differentially expressed over two different conditions (control and disease). In this method, highly correlated nodes are placed into a single module or cluster which are thought to be regulated by the same kind of transcription factors. Therefore, identification of the hub genes of the most significant module can provide insight into the biological significance of that module [74]. Figure 2 describes the gene co-expression network of the most significant co-expressed module (Turquoise module) obtained from WGCNA.

### Identification and further analysis of the hub genes

In order to find out the hub nodes, we analyzed the topological properties of the most significant turquoise module using tYNA (http://tyna.gersteinlab.org/) web interface [33]. Degree and Betweenness Centrality (BC) were selected as the criteria for hub gene selection. Nodes with high degree-low BC value (HC nodes) and nodes with high BC-low degree value (HBLC nodes) were considered as hub nodes. Histone modification data for the hub genes were retrieved from human histone modification database (HHMD, http://202.97.205.78/hhmd/index.jsp) [36]. DIANA-Tarbase [37], miRWalk database [38] and TargetScan database [39] were used to study experimentally validated non-coding miRNA-mediated regulation of hub genes. DIANA-LncBase [40] was used to study lncRNAs associated with these miRNAs. The lncRNAs, which are both experimentally validated and computationally predicted (prediction score ≥ 0.70) are considered in our study. The tissue-specific expression data of eight hub genes were collected from GNF Gene Atlas (http://biogps.org/).

### Construction of regulatory networks

To get a detailed view of the regulatory pattern of the turquoise module, we built a regulatory network comprising genes of the turquoise module, TFs and miRNAs associated with these genes and TFs associated with the miRNAs. The gene-TF information was obtained from TRANSFAC [41]. Information about the miRNAs associated with the genes of the turquoise module was identified from the DIANA-TarBase database [37]. Information about the TFs regulating the transcription of these miRNAs was obtained from the TransmiR database [45]. By combing all these regulatory information, we constructed a TF-miRNA-gene-TF regulatory network (Fig. 3) which represents the four layers of complex regulatory interactions taking place within the most significant WGCNA module. The network was generated by using Cytoscape software [75].

To identify possible lncRNA-mediated regulation of the miRNAs associated with the genes of the turquoise module, we constructed a lncRNA-mediated regulatory network (﻿Figure not shown﻿). The PD-specific miRNAs that were previously found to be associated with the genes of the turquoise module were searched in the lncbase module of DIANA-LncBase [40] to acquire information on the miRNA-lncRNA pair. This database contains experimentally verified and computationally predicted miRNA targets on lncRNAs. The lncRNAs which are both experimentally validated and computationally predicted (prediction score ≥ 0.70) are considered in our study. In order to identify the regulation of these PD-specific miRNAs present in the turquoise module, we constructed a regulatory network involving the TFs, lncRNA and genes associated with these 13 miRNAs (Figure not shown). The TF-miR-lncRNA-gene regulatory network consisted of 44 TFs, 57 lncRNA, 13 miRNAs and 29 genes of the turquoise module (Table 6). The network was generated by using Cytoscape software [75].

### SNP analysis of the highly significant WGCNA module

**Fig. 4:**
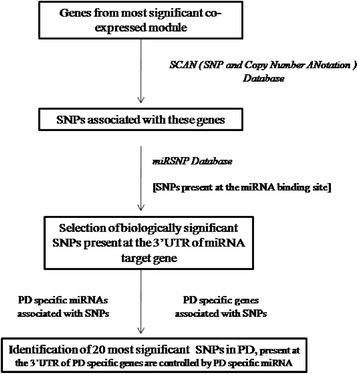
Flowchart for SNP analysis performed in our study

Figure 4 depicts the flowchart for SNP analysis performed in our study. In order to gain insight about the PD-associated SNPs, the 266 genes of the turquoise module were subjected to SNP analysis. SNPs corresponding to these genes were obtained from the online database SCAN (SNP and Copy number ANnotation database; http://www.scandb.org/) [46]. The expression data served in SCAN has been assayed in HapMap (87 CEU and 89 YRI) [76]. CEU represents the human samples of European descent from Utah and YRI represents the Yoruban samples from Ibadan Nigeria. Genes were queried to retrieve information about the relationship between SNPs and genes at user-specified *p*-value thresholds [77]. We chose the SNPs that predict gene expression with *p*-values less than ≤10^−05^ and frequency greater than 0.10. We obtained a huge number of SNPs corresponding to these genes. To identify only the biologically significant SNPs from this huge number of SNPs we sought the SNPs in MirSNP database (http://202.38.126.151/hmdd/mirsnp/search/) [47]. This database identifies SNPs present in the 3′ UTR of miRNA target sites. We obtained 1525 miRNAs corresponding to the SNPs of 266 genes. These 1525 miRNAs were compared with a list of 92 PD-related miRNAs that was obtained through text mining in PubMed and Human MicroRNA Disease Database (HMDD) [78]. We found 82 miRNAs, related to these 92 miRNAs already known in PD. 140 SNPs associated with these 82 miRNAs were considered as the most relevant SNPs in our study, and these were used for further screening.

## Additional files

Additional file 1: Table S1. FatiGO analysis of the turquoise module. S1A - Highly Significant KEGG pathways associated with the turquoise module. S1B - Highly Significant GO Biological Process associated with the turquoise module. (DOCX 12 kb)

Additional file 2: Table S2. Topological properties of the hub genes obtained from the turquoise module. (DOCX 11 kb)

Additional file 3: Table S3. 140 PD-related SNPs identified in our study with their associated chromosome number and PD-related miRNAs. (XLSX 20 kb)

Additional file 4: Table S4. List of lncRNAs associated with the PD-specific miRNAs with their binding type, transcript position, conservation, and MRE sequence. (XLSX 18 kb)

## Abbreviations

BC: Betweenness centrality
DE: Differentially expressed
FFL: Feed Forward Loop
gTF: gene Transcription Factor
HBLC: High Betweenness Low Connectivity
HC: High Connectivity
lncRNA: Long non-coding RNA
miRNA: microRNA
mTF: microRNA Transcription Factor
PD: Parkinson’s disease
SAM: Significance Analysis of Microarray
SNP: Single nucleotide polymorphism
TF: Transcription factor
WGCNA: Weighted Gene Coexpression Network
