# Supplementary material for: Biological networks in Parkinson’s disease: an insight into the epigenetic mechanisms associated with this disease
Source: BMC Genomics. 2017 Sep 12;18:721. doi: 10.1186/s12864-017-4098-3 (PMC5596942; doi:10.1186/s12864-017-4098-3)
Supplement: Supplementary file 1 — FatiGO analysis of the turquoise module. S1A - Highly Significant KEGG pathways associated with the turquoise module. S1B - Highly Significant GO Biological Process associated with the turquoise module. (DOCX 12 kb) [file 12864_2017_4098_MOESM1_ESM.docx]

**Additional file 1**

**Table S1A:** Highly Significant KEGG pathways associated with the turquoise module .

| **Term** | **Name** | **P value** | **Genes** |
| --- | --- | --- | --- |
| hsa05120 | Epithelial cell signaling in Helicobacter pylori infection | 1.71E-04 | NFKBIA,ATP6V0D1,ATP6V1B2,MAP2K4,ATP6V0C,ATP6V1C1 |
| hsa04722 | Neurotrophin signaling pathway | 4.69E-04 | NFKBIA,MAGED1,CALM3,NGFRAP1,YWHAZ,YWHAB,ARHGDIA |
| hsa05012 | Parkinsons Disease Pathway | 5.68E-04 | SNCA,UCHL1,VDAC3,ATP5B,ATP5D,CYCS,PINK1 |

**Table S1B:** Highly Significant GO Biological Process associated with the turquoise module.

| **Term** | **Name** | **P value** | **Genes** |
| --- | --- | --- | --- |
| GO:0007017 | microtubule-based process | 3.47E-08 | NDE1,XPO1,UCHL1,KIF21B,DCTN3,PAFAH1B1,TUBA1,MAP2,TUBB4,KIF3C,NEFL,DCTN2,CLASP2,TUBG2,NDEL1,KATNB1,TUBG1 |
| GO:0007018 | microtubule-based movement | 2.83E-07 | NDE1,UCHL1,KIF21B,PAFAH1B1,TUBA1,TUBB4,KIF3C,NEFL,TUBG2,NDEL1,TUBG1 |
| GO:0007268 | synaptic transmission | 7.30E-06 | KCNMB4,SNCA,MYO5A,SYT1,SLC1A4,STXBP1,VDAC3,PAFAH1B1,GRIA1,USP14,RAB11A,AGTPBP1,SCN2B,PINK1,CALB1,NRXN1 |
| GO:0000904 | cell morphogenesis involved in differentiation | 8.52E-06 | CHST3,PARD3,TGFB3,L1CAM,GAP43,UCHL1,STXBP1,SLITRK5,PAFAH1B1,NEFL,TTC3,THY1,NDEL1,NRXN1 |
| GO:0046907 | intracellular transport | 8.57E-06 | NFKBIA,NDE1,XPO1,TGFB3,SERP1,AP3M2,AP3B2,MYO5A,AKAP12,CALM3,UCHL1,PAFAH1B1,YWHAZ,NEFL,AP2M1,RAB11A,TOM1L2,YWHAB,TOMM20,OPTN,ATP5D,NDEL1,KPNA1,RAB6A,KATNB1,SLC25A17 |
