# Supplementary material for: Biological networks in Parkinson’s disease: an insight into the epigenetic mechanisms associated with this disease
Source: BMC Genomics. 2017 Sep 12;18:721. doi: 10.1186/s12864-017-4098-3 (PMC5596942; doi:10.1186/s12864-017-4098-3)
Supplement: Supplementary file 2 — Topological properties of the hub genes obtained from the turquoise module. (DOCX 11 kb) [file 12864_2017_4098_MOESM2_ESM.docx]

**Additional file 2: Table S2.** Topological properties of the hub genes obtained from the turquoise module.

| **High Degree- low BC hub nodes** | **Degree** | **BC value** | **HBLC hub nodes** | **BC value** | **Degree** |
| --- | --- | --- | --- | --- | --- |
| AP3B2 | 262 | 76.34442 | HNRPC | 391.4984 | 144 |
| MAGED1 | 262 | 76.34442 | MAN1C1 | 264.3293 | 155 |
| NSF* | 262 | 76.34442 | HSPA1A* | 132.9869 | 146 |
| STXBP1 | 262 | 76.34442 |  |  |  |
| CYB561* | 261 | 64.57463 |  |  |  |
| AF1Q | 261 | 57.20812 |  |  |  |
| C14ORF78 | 261 | 64.57463 |  |  |  |
| GASP | 261 | 64.57463 |  |  |  |

* Previously associated with PD.
